# Editorial: Targeting DNA damage response to enhance antitumor innate immunity in radiotherapy

**DOI:** 10.3389/fonc.2023.1257622

**Published:** 2023-07-25

**Authors:** Victoria M. Valvo, Emanuele Vitale, Marco Tigano, Rachel Evans, Meredith A. Morgan, Qiang Zhang

**Affiliations:** ^1^ Department of Radiation Oncology, Rogel Cancer Center, University of Michigan, Ann Arbor, MI, United States; ^2^ Cancer Biology Program, University of Michigan, Ann Arbor, MI, United States; ^3^ Laboratory of Translational Research, Azienda USL-IRCCS di Reggio Emilia, Reggio Emilia, Italy; ^4^ MitoCare Center, Department of Pathology and Genomic Medicine, Thomas Jefferson University, Philadelphia, PA, United States; ^5^ Richard Dimbleby Department of Cancer Research, Randall Division & Division of Cancer Studies, Kings College London, London, United Kingdom; ^6^ Translational Medicine, Oncology R&D, AstraZeneca, Cambridge, United Kingdom

**Keywords:** radiotherapy, DNA damage response, immunotherapy, innate immunity, tumor microenvironment

Radiotherapy is a mainstay of cancer treatment that is used to treat approximately half of all cancers (1) with cure rates second only to surgery. The efficacy of radiotherapy has been largely attributed to the direct killing of tumor cells. Yet, recent research efforts highlighted considerable indirect effects of radiation on the tumor microenvironment (TME), especially the immune compartment, with clinical implications. This active field of research has revealed a complex relationship between radiation and the local/systemic immune system, yielding both immunostimulatory and immunosuppressive effects. Mechanistically, radiation creates a pro-immunogenic environment through the direct release of damage associated molecular patterns (DAMPs) during immunogenic cell death (2). Cells that survive after radiation modulate the immune system by: *1)* intracellular sensing of DAMPs by innate immunity sensors such as cGAS/STING and RIG-I-like receptors followed by production of type 1 interferons, and *2)* tumor-associated antigen cross-presentation (3–5). However, these initial immunostimulatory effects are often counterbalanced by immunosuppression. For instance, intracellularly, autophagy and mitophagy contribute to the clearance of immunostimulatory DAMPs (6). In the TME, longer-term immunosuppressive effects are driven by tumor-associated macrophages and myeloid-derived suppressor cells (7, 8). In addition, immune cell repopulation can occur post radiation as the irradiated tissue is driven towards a wound-healing microenvironment (9). Thus, a complex balance of several factors determines whether radiation induces a suppressed or stimulated immune environment. Current efforts are focused on understanding how the interaction between radiation and immunity plays out in the TME, with the goal of designing interventions to promote an immunostimulatory environment.

Shifting the balance toward the immune stimulatory effects of radiation, requires an in-depth knowledge of the biological effects of radiation on the tumor innate immune response and on the different immune cellular compartments. Furthermore, the contribution of tumor specific characteristics, like tumor type and stage, needs also to be considered. In this special edition, Beach et al. review the differential effects of radiation on macrophage populations in the TME. Tumor associated macrophages can be polarized by radiation into anti-inflammatory/pro-tumorigenic macrophages or pro-inflammatory/anti-tumorigenic macrophages depending on the context (Beach et al.). This exemplifies the dual potential of a single immune cell population within the TME to either promote or eradicate tumor cells, depending on factors including radiation dose, the immune profile of the TME, and the tumor type. Further insight regarding the interplay between the tumor and immune response to radiation is described by Gehre et al. Specifically, the authors demonstrate that radioresistant triple negative breast cancer cells upregulate multiple immune checkpoint molecules on their surface compared to radiosensitive cells upon radiation (Gehre et al.). Whether or not radiation leads to immune stimulation is dependent on a combination of factors including tumor intrinsic properties and the broader immune landscape.

Beyond the direct interactions of radiation with tumor cells and intratumoral immune cells, radiation may also have beneficial effects on peripheral immune cells leading to an adaptive immune response. Craig et al. comprehensively review the abscopal effect, a phenomenon whereby radiotherapy efficacy is extended beyond the tumor in the radiation field to tumor(s) outside of the radiation field by engaging a systemic/adaptive immune response. The presence of an abscopal effect has important implications in the context of metastatic and recurrent disease. Although abscopal responses remain rare in clinical settings, there is growing interest in investigating strategies to enhance the presence and consistency of abscopal responses. For instance, a recent study suggested blocking CD47/SIRPα axis increases radiation-induced phagocytosis and immune priming, leading to enhanced systematic tumor control (10, 11).

Therapeutic strategies that enhance anti-tumoral immune responses to radiotherapy such as those targeting the DNA damage and replication stress responses as well as immune checkpoints are currently an intense area of investigation with potential to further improve patient outcomes to radiotherapy. Daley et al. and Jungles et al. provide comprehensive reviews on the biological rationale and current clinical investigation of combining radiation with other treatment modalities in Ewing sarcoma and breast cancer, respectively. For example, several clinical trials are underway to evaluate the combination of PARP inhibitors, radiotherapy, and immunotherapy in breast cancer patients with or without BRCA deleterious mutations.

Inhibitors of the DNA damage response (DDR) are effective radiation sensitizers targeting multiple protective pathways, such as cell cycle checkpoints and DNA repair, that have recently emerged as promising strategies for sensitizing to immunotherapy (12, 13). Combining DDR inhibitors with radiation is an active area of both pre-clinical and clinical research reviewed by Carlsen and El-Deiry and Chan Wah Hak et al. The ability of DDR inhibitors to enhance radiation-induced immune effects including increased type 1 interferon production and immune cell infiltration is highlighted (Chan Wah Hak et al.). Interestingly, inhibition of different DDR targets enhances radiation efficacy with varying magnitudes by synergizing with different pathways of innate immune signaling (14–17). In this Research Topic, Mariampilla et al. describe how ATR inhibition following radiation enhances interferon signaling mediated by cGAS signaling in human lung cancer and osteosarcoma cells. Additional radiosensitizers, including those which target the replication stress response, are being investigated clinically and are reviewed in Zhang et al. Based on the capacity for DDR inhibitors to enhance the immune effects caused by radiation, it is conceivable that these combinations may further sensitize tumor cells to immunotherapy.

Future investigation into the foundational mechanisms behind radiation-induced immune modulation, as well as the synergies with existing treatment modalities, might provide a rationale for leveraging combinatorial strategies in clinical settings aimed at enhancing radiation-induced immune stimulation and sensitization of tumors to immunotherapy. While these concepts are thoroughly covered in the Research Topic, additional work should focus on determining the differential properties of each treatment, alone or in combination, to reveal which settings provide the best clinical outcomes while minimizing toxicity that could arise in the presence of excess systemic inflammation. This will provide clinicians with needed information to accurately match patients with the most effective treatment to ultimately improve the prognosis of the >18 million of new cancer patients diagnosed each year.

## Author contributions

All authors listed have made a substantial, direct, and intellectual contribution to the work and approved it for publication.

## References

[1] Delaney G Jacob S Featherstone C Barton M. The role of radiotherapy in cancer treatment: estimating optimal utilization from a review of evidence-based clinical guidelines. Cancer (2005) 104(6):1129–37. doi: 10.1002/cncr.21324 16080176

[2] Zhou J Wang G Chen Y Wang H Hua Y Cai Z. Immunogenic cell death in cancer therapy: Present and emerging inducers. J Cell Mol Med (2019) 23(8):4854–65. doi: 10.1111/jcmm.14356 PMC6653385 31210425

[3] Harding SM Benci JL lrianto J Discher DE Minn AJ and Greenberg RA. Mitotic progression following DNA damage enables pattern recognition within micronuclei. Nature (2017) 548(7668):466–70. doi: 10.1038/nature23470 PMC5857357 28759889

[4] Golden EB Marciscano AE and Formenti SC. Radiation therapy and the in situ vaccination approach. Int J Radiat Oncol Biol Phys (2020) 108(4):891–8. doi: 10.1016/j.ijrobp.2020.08.023 32800803

[5] Jagodinsky JC and Morris ZS. Priming and propagating anti-tumor immunity: focal hypofractionated radiation for in situ vaccination and systemic targeted radionuclide theranostics for immunomodulation of tumor microenvironments. Semin Radiat Oncol (2020) 30(2):181–6. doi: 10.1016/j.semradonc.2019.12.008 PMC7286051 32381297

[6] Yamazaki T Kirchmair A Sato A Buque A Rybstein M Petroni G. Mitochondrial DNA drives abscopal responses to radiation that are inhibited by autophagy. Nat Immunol (2020) 21(10):1160–71. doi: 10.1038/s41590-020-0751-0 32747819

[7] Laoui D Van Overmeire E De Baetselier P Van Ginderachter JA Raes G. Functional relationship between tumor-associated macrophages and macrophage colony-stimulating factor as contributors to cancer progression. Front Immunol (2014) 5:489. doi: 10.3389/fimmu.2014.00489 25339957 PMC4188035

[8] Deng L Liang H Burnette B Beckett M Darga T Weichselbaum RR. Irradiation and anti-PD-L1 treatment synergistically promote antitumor immunity in mice. J Clin Invest (2014) 124(2):687–95. doi: 10.1172/JCI67313 PMC3904601 24382348

[9] Cytlak UM Dyer DP Honeychurch J Williams KJ Travis MA lllidge TM. Immunomodulation by radiotherapy in tumour control and normal tissue toxicity. Nat Rev Immunol (2022) 22(2):124–38. doi: 10.1038/s41577-021-00568-1 34211187

[10] Hsieh RC Krishnan S Wu RC Boda AR Liu A Winkler M. ATR-mediated CD47 and PD-L1 up-regulation restricts radiotherapy-induced immune priming and abscopal responses in colorectal cancer. Sci Immunol (2022) 7(72):eabl9330. doi: 10.1126/sciimmunol.abl9330 35687697 PMC9373855

[11] Son J Hsieh RC Lin HY Krause KJ Yuan Y Biter AB. Inhibition of the CD47-SIRPalpha axis for cancer therapy: A systematic review and meta-analysis of emerging clinical data. Front Immunol (2022) 13:1027235. doi: 10.3389/fimmu.2022.1027235 36439116 PMC9691650

[12] Parsels LA Engelke CG Parsels J Flanagan SA Zhang Q Tanska D. Combinatorial efficacy of olaparib with radiation and ATR inhibitor requires PARP1 protein in homologous recombination-proficient pancreatic cancer. Mol Cancer Ther (2021) 20(2):263–73. doi: 10.1158/1535-7163.MCT-20-0365 PMC7867626 33268569

[13] Bryant HE Schultz N Thomas HD Parker KM Flower D Lopez E. Specific killing of BRCA2-deficient tumours with inhibitors of poly(ADP-ribose) polymerase. Nature (2005) 434(7035):913–7. doi: 10.1038/nature03443 15829966

[14] Zhang Q Green MD Lang X Lazarus J Parsels JD Wei S. Inhibition of ATM increases interferon signaling and sensitizes pancreatic cancer to immune checkpoint blockade therapy. Cancer Res (2019) 79(15):3940–51. doi: 10.1158/0008-5472.CAN-19-0761 PMC6684166 31101760

[15] Wang W McMillan MT Zhao X Wang Z Jiang L Karnak D. DNA-PK inhibition and radiation promote antitumoral immunity through RNA polymerase III in pancreatic cancer. Mol Cancer Res (2022) 20(7):1137–50. doi: 10.1158/1541-7786.MCR-21-0725 PMC9262824 35348737

[16] Feng X Tubbs A Zhang C Tang M Sridharan S Wang C. ATR inhibition potentiates ionizing radiation-induced interferon response via cytosolic nucleic acid-sensing pathways. EMBO J (2020) 39(14):e104036. doi: 10.15252/embj.2019104036 32484965 PMC7361286

[17] Tigano M Vargas DC Tremblay-Belzile S Fu Y Sfeir A. Nuclear sensing of breaks in mitochondrial DNA enhances immune surveillance. Nature (2021) 591(7850):477–81. doi: 10.1038/s41586-021-03269-w 33627873

