# Corrigendum: Editorial: Targeting DNA damage response to enhance antitumor innate immunity in radiotherapy

**DOI:** 10.3389/fonc.2024.1424895

**Published:** 2024-06-13

**Authors:** Victoria M. Valvo, Emanuele Vitale, Marco Tigano, Rachel Evans, Meredith A. Morgan, Qiang Zhang

**Affiliations:** ^1^ Department of Radiation Oncology, Rogel Cancer Center, University of Michigan, Ann Arbor, MI, United States; ^2^ Cancer Biology Program, University of Michigan, Ann Arbor, MI, United States; ^3^ Laboratory of Translational Research, Azienda USL-IRCCS di Reggio Emilia, Reggio Emilia, Italy; ^4^ MitoCare Center, Department of Pathology and Genomic Medicine, Thomas Jefferson University, Philadelphia, PA, United States; ^5^ Richard Dimbleby Department of Cancer Research, Randall Division & Division of Cancer Studies, Kings College London, London, United Kingdom; ^6^ Translational Medicine, Oncology R&D, AstraZeneca, Cambridge, United Kingdom

**Keywords:** radiotherapy, DNA damage response, immunotherapy, innate immunity, tumor microenvironment

In the published article, an author name was incorrectly written as Victoria Valvo. The correct spelling is Victoria M. Valvo.

The authors apologize for this error and state that this does not change the scientific conclusions of the article in any way. The original article has been updated.

